# Atrial Mass Versus Thrombus

**DOI:** 10.4274/balkanmedj.galenos.2019.2019.11.79

**Published:** 2020-04-10

**Authors:** Yugandhara Kate, Masood Pasha Syed, Adhirath Doshi, Shekhar Patil, Deepti Kumar

**Affiliations:** 1 Clinic of Internal Medicine, Saint Vincent Hospital, Worcester, Massachusetts; 2 Clinic of Cardiology, Saint Vincent Hospital, Worcester, Massachusetts

An 84-year-old woman with a past medical history of significant hyperlipidemia, hypertension, impaired glucose tolerance, and asthma along with progressive shortness of breath for 2 weeks and exacerbating swelling of lower extremity was presented to the hospital. She had visited her primary care physician for the same symptoms and was administered with furosemide before the hospital presentation. Her lower extremity swelling and other symptoms did not show any improvement with furosemide administration, which prompted her visit to the emergency room.

Upon presentation, new-onset atrial fibrillation showing a heart rate of 170 beats per min was observed. Further physical examination revealed irregular heart rhythms with bibasilar crackles on auscultation. Initial lab results were normal except for the following values: hemoglobin of 11.3, pro B-type Natriuretic Peptide (pro-BNP) of 5197, and INR of 1.1. She was administered with oral diltiazem, which failed to improve her heart rate. Eventually, intravenous administration of diltiazem was started. She was also administered with IV digoxin and was further initiated on anticoagulation with enoxaparin.

As part of the cardiology workup, she underwent a transthoracic echocardiogram showing left ventricular ejection fraction of 40%–45% and mild-to-moderate global hypokinesis. The right ventricle was dilated with mildly reduced systolic function, biatrial enlargement with severe tricuspid regurgitation, and pulmonary artery systolic pressure of 45 mmHg. A hypermobile mass, which measured 2.39 cm×1.1 cm, was also observed in the left atrium. This mass was attached to the interatrial septum. The initial differential diagnosis included myxoma, thrombus, or other tumors. Intravenous administration of heparin was started preemptively, whereas transesophageal echocardiogram (TEE) was being planned to rule out the large thrombus. TEE confirmed a large mass in the left atrium attached to the septum by a thin stalk measuring up to 4.3 cm in length [Supplementary Figure 1, Supplementary Video 1(2D), Supplementary Video 2 (3D)]. Spontaneous contrast was observed in the L atrial appendix and no thrombus was detected. The patient underwent subsequent surgical excision of the mass and ligation of the left atrial appendage using an atrial clip device. Pathological examination confirmed the findings of left atrial myxoma. Verbal consent was obtained from the patient.

We want to highlight the diagnostic dilemma presented in this scenario. Acute presentation of dyspnea (seen more with the left-sided myxoma as compared with the right-sided myxoma) (1,2) in the setting of new-onset atrial fibrillation can have a broader etiology with varied implications. A mass can be easily mistaken as a thrombus, (3) which changes the line of management, especially if cardioversion is being planned. In our patient, TEE was instrumental in the characterization of the atrial mass, which further guided the surgical management for the mass over the medical management of the thrombus (4,5).

## Figures and Tables

**Figure 1:**
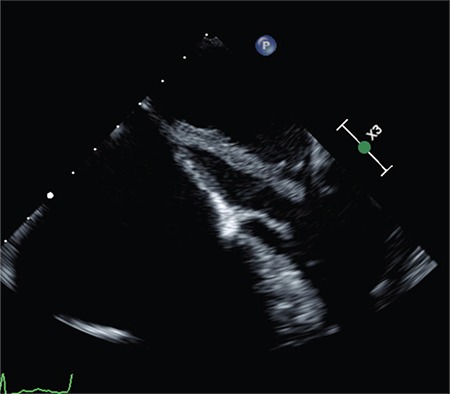
Transesophageal echocardiogram showing a mass of 4.3 cm originating from the left atrium and protruding into the mitral orifice.
